# Summer Complaints—Prophylaxis

**Published:** 1897-08

**Authors:** A. L. Benedict

**Affiliations:** Buffalo, N. Y.; Professor of Physiology and Digestive Diseases, Dental Department, University of Buffalo


### SUMMER COMPLAINT—PROPHYLAXIS.

BY A. L. BENEDICT, A. M., M. D., BUFFALO, N. Y.

Professor of Physiology and Digestive Diseases, Dental Department, University of Buffalo.

When the editor of the *Fortnightly* asked me to contribute an article to a symposium on summer complaint, I was forced to explain that, notwithstanding the nature of my practice, cases of this kind were quite rare and that I was much better prepared to discuss the reasons for their infrequency in Buffalo than to throw light on the disease itself. In referring to the matter in this narrow way, it is still possible to draw generally useful conclusions from local facts.

But first, that there may be no confusion from differences in the use of terms, it may be well to define summer complaint and, incidentally, to explain that, even in Buffalo, we are not devoid of opportunities for studying this pest of infancy. Summer complaint is due to fermentation in the alimentary tract, usually of food, though, in some instances, mucus, cast-off epithelium, etc., seem to be the nidus of the trouble. Precisely the same chemical and bacteriological processes may take place at other seasons, but sources of infection are, naturally, more abundant in the heated term. While, in a general sense, a germ disease, there is no one specific germ, and it seems probable that other micro-organisms than the true bacteria, may be active as poison producers. As to the actual presence of yeast, etc., there is no question. In adults this process usually produces nothing more tangible than a hyperæmia. In young children, a greater or less area of mucous membrane is actively inflamed; in other words, there is gastritis, a duodenitis, a gastro-enteritis, an entero-colitis, or a more or less general involvement of the alimentary canal from the cardia to the anus. Thus, the technical terminology, the exact symptoms and the local treatment must vary according to the localization of the process. In children, the febrile reaction marking the inflammatory trouble is conspicuous; in adults, fever is usually slight. The organic process of fermentation—of which the really important ones are scarcely known to us as yet—are absorbed into the blood and excreted largely by the kidneys, so that the urine becomes more toxic than usual. Especially in adults, there may be direct irritation of the urinary passages, amounting sometimes to quite a typical cystitis. I may mention one such case, though occurring in the spring, in which the bladder symptoms so far masked the gastro-intestinal trouble that my first impression was that the case was quite out of my domain. There was a dilatation of the stomach of moderate grade, which had caused little annoyance. The use of improper food and excessive drinking of ice-water while traveling had precipitated the attack. The urine was scanty, contained indican in excess, some blood and mucus, and bladder epithelium. There was intense vesical tenesmus. There was no account of previouz bladder trouble and the only possible local cause which could be elicited, even by leading questions, was rather excessive indulgence, but with no probability of venereal infection.

Summer complaint in children is very apt to complicate or to be complicated by pneumonia. Milk and its preparations are especially liable to contamination, and it may be that almost all cases are especially tyrocoxicon irritations or inflammations. While summer complaint is notorious in bottle-fed children, it must always be borne in mind that a filthy breast, an unlean mouth or any other failure to preserve asepsis, may overcome the beneficial influence of breast-milk, that artificial feeding may be accomplished safely by a well-informed physician, provided he has the co-operation of an intelligent and conscientious nurse or mother, and that good artificial food is infinitely better than milk from a diseased or enfeebled mother.

Overcrowding, absence of sunlight, defective ventilation, insufficient garbage service, lack of inspection of foods, especially of milk, fruits and vegetables, bad water supply, are the chief predisposing factors in the production of summer complaint, that lie within the scope of the municipality. Aside from natural conditions that make the avoidance of these evils difficult, climate may also effect local differences in predisposition. The other point deserves mention—popular recognition of the fact that germs are neither Republican nor Democratic in their activity.

Excessive heat is an evil from which Buffalo is exceptionally free, on account of the cooling action of Lake Erie. The prevalence of west winds throughout most of the country probably explains the fact that Chicago, situated on Lake Michigan, and the Atlantic cities lack this favorable element. Although Lake Erie acts as a refrigerator for Buffalo, and also as a wall against tornadoes and all sorts of meteriological errors, it does not cause the insufferable "humidity" of Boston and other places. True, the air is damp and abominably irritating in the spring, but there is none of the depressing excess of moisture that deserves a word of more syllables than dampness. Once in five or ten years, some suffering and much grumbling is caused by a temperature of 90° or 95° continued for a few hours, but, as a rule, the nights are cool enough to require blankets and a temperature over 75° is exceptional. But it is noticeable that parts of the municipalty remote from the lake, are appreciably warmer, and this is especially true of the parts of "Greater Buffalo" not yet included in the municipality, as the Tonawandas and Niagara Falls.—*Medical Fortnightly.*

